# EyeGPT for Patient Inquiries and Medical Education: Development and Validation of an Ophthalmology Large Language Model

**DOI:** 10.2196/60063

**Published:** 2024-12-11

**Authors:** Xiaolan Chen, Ziwei Zhao, Weiyi Zhang, Pusheng Xu, Yue Wu, Mingpu Xu, Le Gao, Yinwen Li, Xianwen Shang, Danli Shi, Mingguang He

**Affiliations:** 1 School of Optometry The Hong Kong Polytechnic University Hong Kong China; 2 State Key Laboratory of Ophthalmology Zhongshan Ophthalmic Center, Sun Yat-sen University Guangdong Provincial Key Laboratory of Ophthalmology and Visual Science, Guangdong Provincial Clinical Research Center for Ocular Diseases Guangzhou China; 3 Department of Ophthalmology Shanghai General Hospital (Shanghai First People’s Hospital), School of Medicine Shanghai Jiao Tong University Shanghai China; 4 National Clinical Research Center for Eye Diseases Shanghai China; 5 Research Centre for SHARP Vision (RCSV) The Hong Kong Polytechnic University Hong Kong China; 6 Centre for Eye and Vision Research (CEVR) 17W Hong Kong Science Park Hong Kong China

**Keywords:** large language model, generative pretrained transformer, generative artificial intelligence, ophthalmology, retrieval-augmented generation, medical assistant, EyeGPT, generative AI

## Abstract

**Background:**

Large language models (LLMs) have the potential to enhance clinical flow and improve medical education, but they encounter challenges related to specialized knowledge in ophthalmology.

**Objective:**

This study aims to enhance ophthalmic knowledge by refining a general LLM into an ophthalmology-specialized assistant for patient inquiries and medical education.

**Methods:**

We transformed Llama2 into an ophthalmology-specialized LLM, termed EyeGPT, through the following 3 strategies: prompt engineering for role-playing, fine-tuning with publicly available data sets filtered for eye-specific terminology (83,919 samples), and retrieval-augmented generation leveraging a medical database and 14 ophthalmology textbooks. The efficacy of various EyeGPT variants was evaluated by 4 board-certified ophthalmologists through comprehensive use of 120 diverse category questions in both simple and complex question-answering scenarios. The performance of the best EyeGPT model was then compared with that of the unassisted human physician group and the EyeGPT+human group. We proposed 4 metrics for assessment: accuracy, understandability, trustworthiness, and empathy. The proportion of hallucinations was also reported.

**Results:**

The best fine-tuned model significantly outperformed the original Llama2 model at providing informed advice (mean 9.30, SD 4.42 vs mean 13.79, SD 5.70; *P*<.001) and mitigating hallucinations (97/120, 80.8% vs 53/120, 44.2%, *P*<.001). Incorporating information retrieval from reliable sources, particularly ophthalmology textbooks, further improved the model's response compared with solely the best fine-tuned model (mean 13.08, SD 5.43 vs mean 15.14, SD 4.64; *P*=.001) and reduced hallucinations (71/120, 59.2% vs 57/120, 47.4%, *P*=.02). Subgroup analysis revealed that EyeGPT showed robustness across common diseases, with consistent performance across different users and domains. Among the variants, the model integrating fine-tuning and book retrieval ranked highest, closely followed by the combination of fine-tuning and the manual database, standalone fine-tuning, and pure role-playing methods. EyeGPT demonstrated competitive capabilities in understandability and empathy when compared with human ophthalmologists. With the assistance of EyeGPT, the performance of the ophthalmologist was notably enhanced.

**Conclusions:**

We pioneered and introduced EyeGPT by refining a general domain LLM and conducted a comprehensive comparison and evaluation of different strategies to develop an ophthalmology-specific assistant. Our results highlight EyeGPT’s potential to assist ophthalmologists and patients in medical settings.

## Introduction

Ophthalmic diseases pose significant concerns for public health [1]. However, shortages of professionals and inefficiencies in primary eye care systems often funnel patients into overcrowded tertiary centers. This results in extended wait times and unaddressed postconsultation questions, frequently requiring additional face-to-face appointments [2]. These challenges can be attributed to the limited ophthalmic knowledge among patients and the limited experience in eye care among primary health care providers [3]. Therefore, there is a pressing need to enhance ophthalmic health education for both patients and primary health care providers. However, relying solely on manpower to address these issues presents further challenges, particularly as the rate of population aging continues to outpace the growth rate of ophthalmologists.

Large language models (LLMs) have recently emerged as powerful tools to alleviate these burdens and streamline clinical flow with the capability of understanding and generating human-like text [4]. In ophthalmology, LLMs show promise both for ophthalmic certification exams [5] and interpreting imaging reports across various linguistic environments [6,7]. However, there are several limitations to existing LLMs. First, there are challenges with addressing specialized ophthalmology knowledge for general LLMs. Previous research has demonstrated the suboptimal performance of ChatGPT in ophthalmology, with only 15.4% of the responses graded as completely accurate in vitreoretinal disease [8]. Even with GPT-4, which currently exhibits the greatest capability, nonnegligible instances of misinformation occur, with only 30.6%, 21.5%, and 55.6% of responses about ocular multimodal images considered accurate, highly usable, and harmless, respectively [9]. A critical factor underlying these shortcomings is the model’s insufficient grasp of specialized knowledge, particularly in handling medical abbreviations and jargon within highly specialized domains [5]. Therefore, there is a need to design a dedicated model trained on clinically relevant domain data. Second, it is widely recognized that LLMs occasionally generate inaccurate and misleading statements (hallucinations), which can potentially lead to medical errors. Fine-tuning with professional data can somewhat mitigate hallucinations, but the model can still produce them when faced with unfamiliar input [10]. Therefore, additional solutions are required. Third, there is a noticeable absence of comprehensive evaluations for LLMs in ophthalmology. Although previous studies have explored the ophthalmic question-answering (QA) capabilities of LLMs, the majority have been limited to multiple-choice formats [5,11-13]. Although a few studies have used open-ended questions to evaluate the performance of LLMs, they lack detailed categorization of the questions and primarily focus on scattered aspects such as accuracy, comprehensiveness, or safety [14,15]. Consequently, a comprehensive evaluation framework is urgently needed to test ophthalmology-related LLMs and compare their responses with those provided by certified ophthalmologists.

Recognizing this, we aimed to develop an artificial intelligence (AI) assistant, namely EyeGPT, to meet the specific informational needs in ophthalmic clinical and educational scenarios. By leveraging Llama2, a flexible and scalable open-source LLM known for its impressive performance in medicine [16-18], we infused the model with a granular level of ophthalmic expertise through role-playing, fine-tuning, and retrieval-augmented generation (RAG). The resultant model, EyeGPT, was evaluated for its efficacy in patient consultations and medical education. This work provides valuable insights into building and evaluating ophthalmic assistants, paving the way for the next generation of AI-assisted ophthalmic practice.

## Methods

### Ethical Considerations

**Figure 1:**
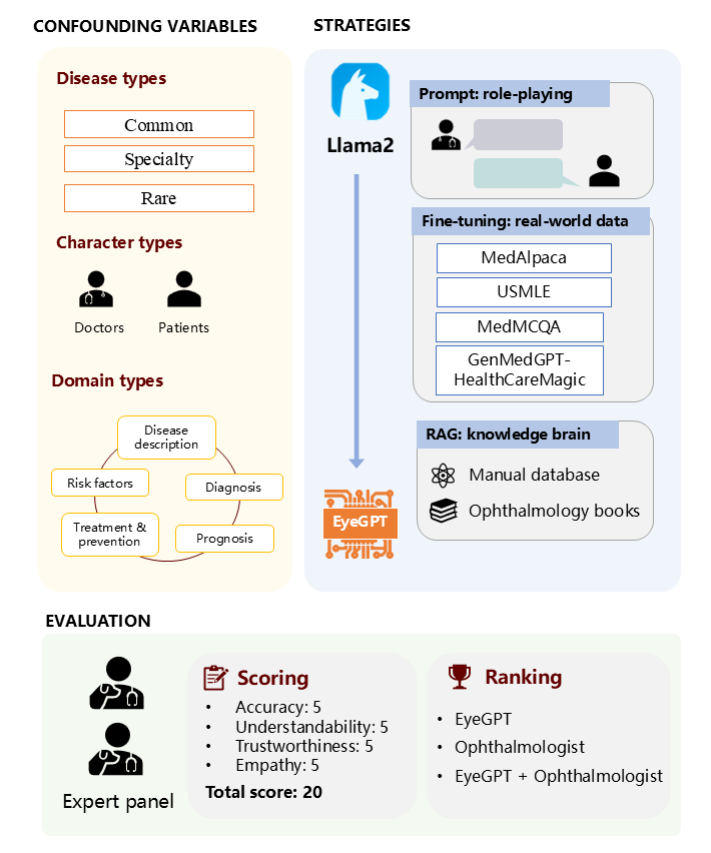
Overview of this study. GPT: generative pre-trained transformer; MCQA: multiple-choice question answering; RAG: retrieval-augmented generation; USMLE=United States Medical Licensing Examination.

The study overview is presented in Figure 1. Our research protocol adhered to the principles of the Helsinki Declaration. The study was approved by the Institutional Review Board of the Hong Kong Polytechnic University (number: HSEARS20240202004). This research involves publicly available data. We ensured that the data were deidentified and all private information was removed. Informed consent was unnecessary as the publicly available data do not contain identifiable information.

### Development of EyeGPT

#### Base Model

We used Meta’s Llama2 as the base model in our study, which was trained on 2 trillion tokens from publicly accessible data [19]. We used the Llama2-7b-chat model, which was additionally fine-tuned on publicly available instruction data sets and over 1 million human annotations, thus having basic conversation skills [20]. To inject professional ophthalmic knowledge into the model, we did experiments successively under the scenarios described in the following paragraphs.

#### Role-Playing

In generative AI, the engineering technique known as “role-playing” involves directing LLMs to “embody” or “imitate” specific roles for improved results [21]. To enable the LLM to generate more relevant and empathetic responses, we assigned it the role of an “ophthalmologist” and the user the dual roles of a “patient” and “medical student.” This was achieved by giving the following instructions: “Suppose you are an ophthalmologist, and you need to answer the patient’s question with care/student’s question with patience.”

#### Fine-Tuning

To inject domain-specific knowledge and make Llama2 more proficient in capturing ophthalmic terminologies and logical reasoning, we trained it on domain-specific data sets, including MedAlpaca [22], GenMedGPT-HealthCareMagic [23], MedMCQA [24], and the United States Medical Licensing Examination (USMLE). Processing of the USMLE data followed the method proposed by Jin et al [25]. The data sets were filtered to remove conversations of little practical significance and responses with errors. We used instruction tuning [26] to align the model with task-specific user objectives, enhance model controllability, and ensure rapid domain-specific adaptation. For data sets initially designed for multiple-choice QA, we automatically added an instruction at the beginning: “Answer the multiple-choice question.” For our specific task, we filtered out nonophthalmology data with eye-related keywords. Multimedia Appendix 1 presents the characteristics of the filtered data sets, and Multimedia Appendix 2 lists the keywords we used.

The final data set comprised 83,919 samples, with 81,919 used for training and 2000 used for validation. We used low-rank adaptation (LoRA) [27] to fine-tune the Llama2-7B model by adding a low-rank matrix while keeping the original parameters frozen, aiming to complement the original weight matrices of the model. The models were fine-tuned using 3*V100 GPUs with a batch size of 24, learning rate of 0.00003, maximum sequence length of 512 tokens, and warm-up ratio of 0.03. For LoRA-specific hyperparameters, the rank of low-rank factorization was 8, the scaling factor for the rank was 16, and the dropout was 0.05. Specifically, we performed 3 types of fine-tuning: Fine-tune 1 (2000 iterations), Fine-tune 2 (3500 iterations), and Fine-tune 3 (10,000 iterations). The entire training process took approximately 11 hours to complete.

#### Retrieval-Augmented Generation

LLMs may produce potential inaccuracies responses (hallucinations) to questions [28], which is unacceptable in the medical field. However, the accuracy of these models could be significantly improved if they could generate responses based on a reliable knowledge database. Here, to further improve the performance of EyeGPT, we introduced the external knowledge corpus of medical books and a manual database.

For the medical books, we used 14 specialized ophthalmology textbooks that cover a wide range of comprehensive ophthalmic knowledge, including general ophthalmology, optometry, retinal diseases, and more [29-31]. Please refer to Multimedia Appendix 3 for the specific textbook list.

We manually built a database (sample shown in Multimedia Appendix 4) containing information on diseases, symptoms, medical tests and treatment procedures, and potential medications. This database, sourced from the open-access web and research papers, serves as an external and offline knowledge corpus for EyeGPT. It can be continually updated without model retraining and may provide more up-to-date information than textbooks.

To leverage external knowledge, we adopted the LangChain framework’s information retrieval techniques. The “all-MiniLM-L6-v2” [32] open-source embedding model was used to map text into vector space. We used the “RecursiveCharacterTextSplitter” [33] to segment the text for efficient retrieval, with a chunk size set to 1024 characters. Roughly 2 segments are retrieved from the vector storage for each response. In addition, we constructed a retriever with Facebook AI Similarity Search (FAISS) [34] based on the segmented documents and established a conversational retrieval chain that seamlessly integrated our EyeGPT with the external database through LangChain.

### Evaluation

#### Overview of the Evaluation

To assess the professional performance of various EyeGPT variants, namely (1) original (Llama2), (2) role-play (original plus role-play), (3) fine-tune 1-3 (fine-tuned model versions 1-3 plus role-play), (4) role-play+book (role-play plus book retrieval), (5) role-play+database (role-play plus manual database retrieval), (6) best fine-tune+book (the best fine-tuned model plus book retrieval), (7) best fine-tune+database (the best fine-tuned model plus manual database retrieval), our ophthalmology expert panel curated a set of 120 ophthalmic care-related questions based on their clinical expertise. We followed the user-centered evaluation approach proposed by Abbasian et al [35], considering the following 3 key factors: disease type, character type, and domain type. Disease type covered a wide range of medical conditions from various subspecialties, including common, specialty, and rare diseases, resulting in 12 disease categories such as myopia, retinal detachment, and Stickler syndrome (refer to Multimedia Appendix 5 for the detailed disease list). Character types included patients and medical students representing potential EyeGPT users. Domain types were divided into 5 topics: disease description, risk factors, diagnosis, treatment and prevention, and prognosis. We conducted the evaluations manually, including an independent evaluation of different EyeGPT variants, best-ranked comparisons for evaluating human-machine performance, and error analysis of the machine.

#### Independent Evaluation

This evaluation was designed to compare the performance of various optimization strategies of the EyeGPT variants and identify the best-performing one. Two board-certified ophthalmologists independently conducted manual assessment using a 5-point scale to assess the responses of each variant. The evaluation focused on the following 4 aspects: accuracy, understandability, trustworthiness, and empathy [35]. The detailed grading scale is presented in Multimedia Appendix 6. The scale ranged from 1 (strongly disagree) to 5 (strongly agree), with the average score from the 2 evaluators recorded as the score for each response aspect. The maximum score for each aspect was 5, and these scores were summed to obtain the final score for each response, with a maximum possible score of 20.

To evaluate the effectiveness of different optimization strategies in mitigating hallucinations, we defined answers with accuracy scores below 4 as containing hallucinations in our study. To ensure the evaluators could not identify the source of the responses, all generated responses were formatted as plain text, concealing any model-specific features. These responses were then randomly shuffled and mixed before being presented to the evaluators.

The evaluation was conducted in 2 rounds with a 1-month washout period to mitigate residual effects [36,37]. In the first round, we compared models using different fine-tuning approaches, including original, role-play, and fine-tune 1-3. The goal was to determine the best fine-tuning model for the subsequent RAG. In the second round, we compared models using different RAG strategies based on the best-performing fine-tuned model selected from the first round. These models included best fine-tune (the best fine-tuned model from round 1), role-play+database, best fine-tune+database, role-play+book, and best fine-tune+book.

#### Best-Ranked Comparison

After independently evaluating the different EyeGPT variants, we identified the best-performing system. To assess if EyeGPT can match ophthalmologists’ expertise and offer them assistance, we conducted a human-machine best-ranked comparison. This evaluation method, inspired by that of Tu et al [38], aimed to efficiently assess answers comprehensively, reducing the need for assessors to delve into every detail and thereby minimizing subjectivity.

We invited 2 junior ophthalmologists (with 1-3 years of clinical experience) to answer the 120 questions with and without the aid of EyeGPT. The answers from different groups (EyeGPT, unassisted ophthalmologist, and EyeGPT+ophthalmologist) were evaluated by 2 senior ophthalmologists (with over 3 years of clinical experience) who were unaware of the sources, and the presentation order was randomized. Raters were asked to rank the 3 answers based on their clinical judgment across 4 dimensions, without the option of declaring a tie. In cases of disagreement, an ophthalmology expert (with over 10 years of clinical experience) reviewed the case until consensus was reached. The final result was recorded as the proportion of responses from different sources ranked as the best.

#### Error Analysis

To further investigate the quality of EyeGPT answers and identify areas for improvement, we conducted an error analysis on the best-performing EyeGPT model. The quality of the EyeGPT-generated QA pairs was evaluated by 2 board-certified ophthalmologists based on their expert judgment. The analysis focused on identifying occurrences of unrelated information, factual errors, incomplete information, and faulty logic [39].

### Statistical Analysis

Statistical analyses were conducted using R (Version 4.3.1). The Mann-Whitney *U* test was used to compare the scores of the 2 models in the independent evaluation. When creating the bar chart, we compared the performance of the base model (Llama 2 or best fine-tune) with the most competitive optimization model in the same round to display statistically significant differences on the chart. The score for each answer in the independent evaluation was based on the average score from 2 raters. The scoring criteria used in the bar chart were as follows: strongly disagree (1 to <2), agree (2 to <3), neutral (3 to <4), approve (4 to <5), strongly agree (5). For subgroup analysis based on different confounding variables, the Kruskal-Wallis test and Mann-Whitney *U* test were used, depending on the number of comparison groups. Cohen kappa was calculated to determine the agreement among raters [40]. *P* values <.05 were considered statistically significant.

## Results

### Comparative Study of Model Construction Strategies

#### Overall Performance

In the first round of evaluation, the total scores for the original, role-play, and fine-tune 1-3 models were 9.30, 12.79, 12.95, 12.83, and 13.79, respectively. All optimized models significantly outperformed the original model in accuracy, understandability, trustworthiness, and empathy, with fine-tune 3 performing the best. For the different fine-tuning variants, we observed that, as the number of iterations increased, the evaluation loss on the test data decreased (refer to Multimedia Appendix 7) and the model performance improved. In the subsequent comparison of RAG strategies, the best fine-tune+book model scored the highest, at 15.14, outperforming other strategies, as elaborated in Multimedia Appendix 8**.** To ensure reliability, we compared the scores of fine-tune 3 (named best fine-tune in round 2) across 2 rounds. We found no statistically significant difference between the scores of the 2 rounds (*P*=.11). Inter-rater reliability in 2 rounds of independent evaluation was confirmed, with kappa values ranging from 0.611 to 0.872, indicating substantial agreement among raters (Multimedia Appendix 9). For illustrative examples of the varied grades of responses from the independent evaluation, see Multimedia Appendix 10.

**Figure 2:**
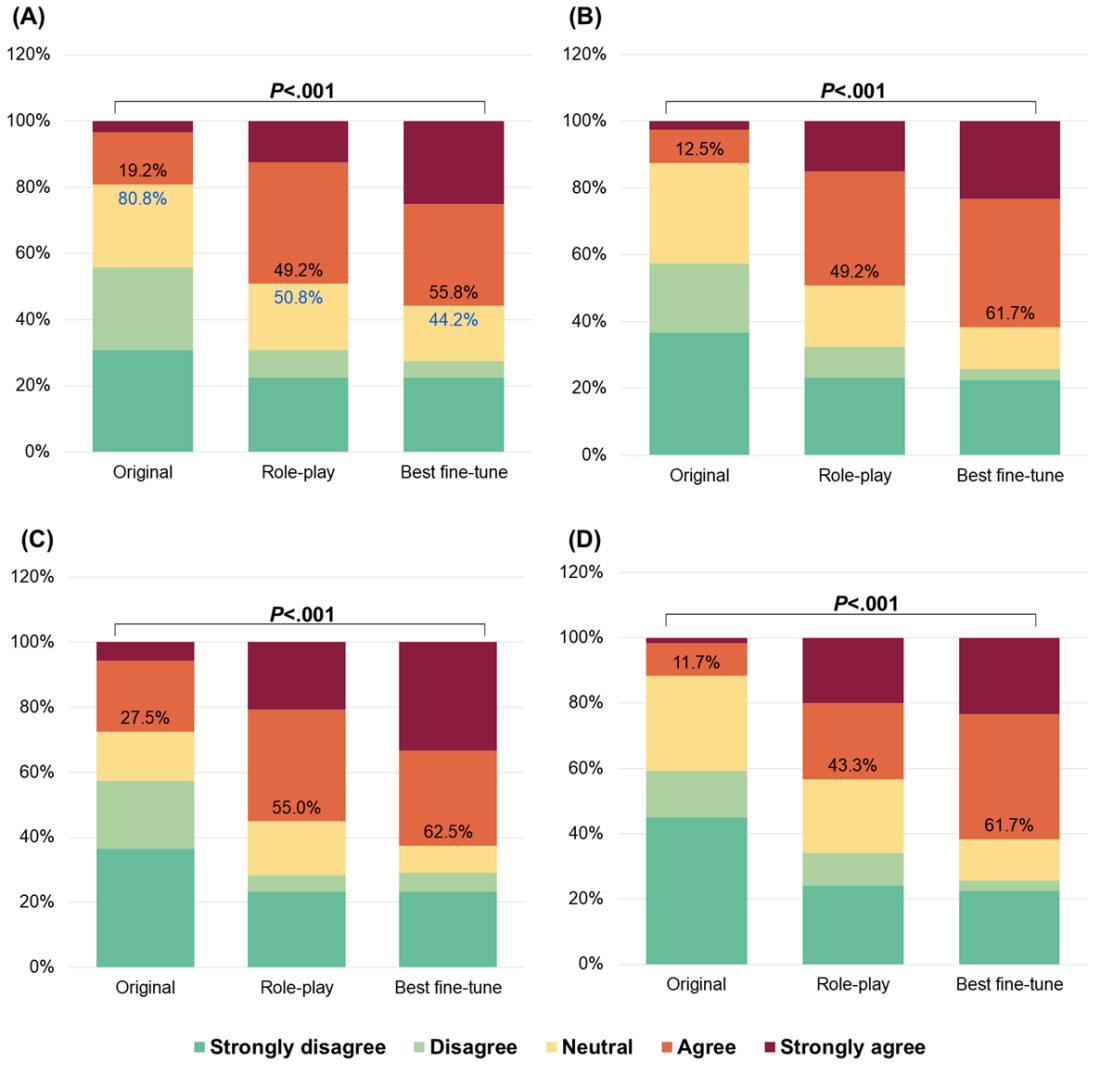
Performance in terms of (A) accuracy, (B) understandability, (C) trustworthiness, and (D) empathy of the different models in round 1 of the human evaluation, with the percentage of good responses (strongly agree and agree) indicated by the black numbers, the percentage of hallucinations indicated by the blue numbers, and significance determined using Mann-Whitney *U* tests.

**Figure 3:**
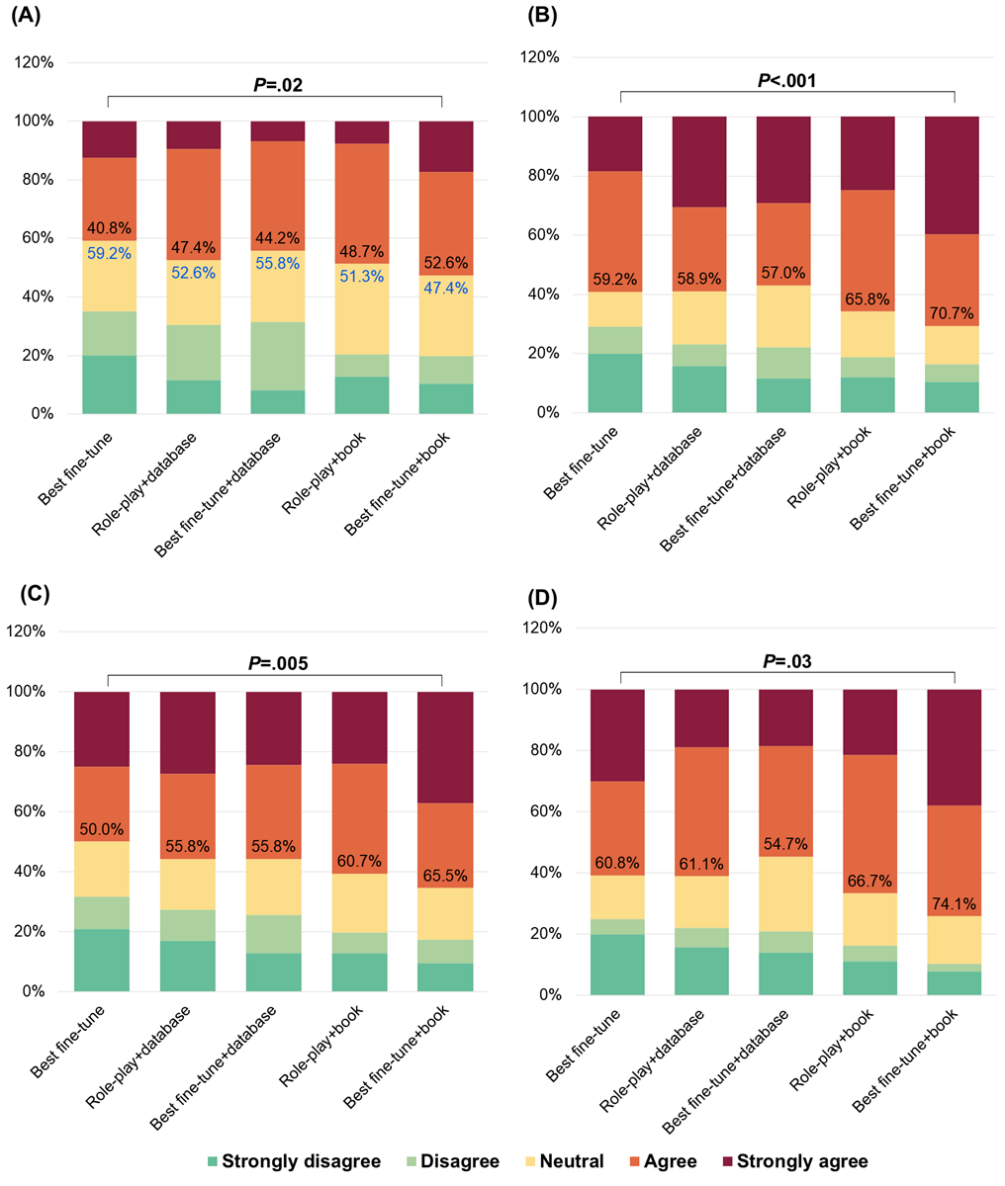
Performance in terms of (A) accuracy, (B) understandability, (C) trustworthiness, and (D) empathy of the different models in round 2 of the human evaluation, with the percentage of good responses (strongly agree and agree) indicated by the black numbers, the percentage of hallucinations indicated by the blue numbers, and significance determined using Mann-Whitney *U* tests.

Figure 2 demonstrates that more than one-half (accuracy: 67/120, 55.8%; understandability: 74/120, 61.7%; trustworthiness: 75/120, 62.5%; empathy: 74/120, 61.7%) of responses from the best fine-tune model were considered “good” responses (rated 4 or above) across all 4 dimensions. Compared with the original model (with an 80.8% [97/120] hallucination rate), the role-play and best fine-tune models mitigated hallucinations, by 30% (36/120) and 36.7% (44/120), respectively. Figure 3 shows that the best fine-tune+book model further enhanced the proportion of “good” responses to the maximum. We compared the performance of the best model in round 1 (fine-tune 3) with the most competitive modified model to check for statistically significant differences. The scores and scoring criteria are the same as in Figure 2. Compared with the best fine-tune model, the best fine-tune+database and best fine-tune+book models further reduced hallucinations by 3.3% (4/120) and 11.7% (14/120), respectively.

#### Subgroup Analysis

We also performed subgroup analysis to further evaluate the model performance under different confounding factors, including subspecialty questions of varying difficulty levels, questions raised by different characters, and question domains.

##### Different Subspecialties

Across all RAG strategies, the models scored higher for common diseases than for specialty and rare conditions (Table 1). For common ophthalmic conditions, the RAG models delivered more precise and contextually relevant information. For more specialized conditions like central serous chorioretinopathy, the best fine-tune model provided general information about its treatment options, while the RAG models offered more specialized responses concerning laser treatment and photodynamic therapy depending on the specific circumstances. For rare conditions like morning glory syndrome, although the best fine-tune model could not generate responses as it mistakenly identified it as “bilateral posterior superior temporal arcade spikes,” the RAG model was able to retrieve relevant information from the external knowledge database and make accurate responses. The best fine-tune model accurately recognized 43% (13/30) of ophthalmic abbreviations. RAG strategies improved this recognition rate, ranging from 60% (18/30) to 83% (25/30) for different models.

**Table 1:** Subgroup analysis of the performance of EyeGPT by subspecialty.

EyeGPT model	Common diseases, mean (SD)^a^	Specialty diseases, mean (SD)^a^	Rare diseases, mean (SD)^a^	*P* value
Best fine-tune^b^	15.79 (4.15)	13.11 (5.40)	10.33 (5.31)	<.001
Role-play+database^c^	15.28 (5.17)	14.18 (5.11)	12.18 (4.88)	.01
Best fine-tune+database^d^	15.45 (4.57)	14.29 (4.89)	12.17 (4.58)	.01
Role-play+book^e^	15.70 (3.94)	12.89 (5.32)	14.66 (4.53)	.02
Best fine-tune+book^f^	17.24 (3.02)	14.08 (5.49)	14.23 (4.42)	.003

^a^Overall response score (the sum of 4 rating dimensions, with a maximum score of 20 representing the best performance).

^b^The fine-tuned model with 10,000 iterations.

^c^Role-play plus manual database retrieval.

^d^The best fine-tuned model plus manual database retrieval.

^e^Role-play plus book retrieval.

^f^The best fine-tuned model plus book retrieval.

##### Different Role-Play Characters

When comparing the influence of the questioner’s assumed identity—patient versus medical student—on model performance, responses to patients consistently scored higher than those of medical students (Table 2). This difference reached statistical significance in the best fine-tune and role-play+database models. However, no significant differences were observed with the best fine-tune+database, role-play+book, and best fine-tune+book models, suggesting that these adjusted models can answer both general patient questions and more specialized queries from medical students.

**Table 2:** Subgroup analysis of the performance of EyeGPT by role-play character.

EyeGPT model	Patients, mean (SD)^a^	Medical students, mean (SD)^a^	*P* value
Best fine-tune^b^	13.45 (5.79)	10.99 (6.32)	.03
Role-play+database^c^	14.67 (5.07)	11.62 (6.55)	.03
Best fine-tune+database^d^	14.52 (4.99)	12.84 (5.34)	.08
Role-play+book^e^	14.85 (4.78)	12.65 (5.85)	.06
Best fine-tune+book^f^	14.44 (6.13)	13.38 (5.83)	.07

^a^Overall response score (the sum of 4 rating dimensions, with a maximum score of 20 representing the best performance).

^b^The fine-tuned model with 10,000 iterations.

^c^Role-play plus manual database retrieval.

^d^The best fine-tuned model plus manual database retrieval.

^e^Role-play plus book retrieval.

^f^The best fine-tuned model plus book retrieval.

##### Different Domains

In the subgroup analysis of EyeGPT’s performance across different domains, there were no statistically significant differences in the scores of disease description, risk factors, diagnosis, treatment and prevention, and prognosis across all models (Table 3).

**Table 3:** Subgroup analysis of the performance of EyeGPT by domain.

EyeGPT model	Disease description, mean (SD)^a^	Risk factors, mean (SD)^a^	Diagnosis, mean (SD)^a^	Treatment and prevention, mean (SD)^a^	Prognosis, mean (SD)^a^	*P* value
Best fine-tune^b^	12.92 (6.51)	12.67 (5.38)	11.81 (6.45)	13.21 (5.12)	9.90 (6.60)	.35
Role-play+database^c^	12.98 (6.57)	12.73 (6.00)	13.73 (5.38)	13.53 (4.55)	11.48 (6.26)	.49
Best fine-tune+database^d^	15.14 (4.76)	12.08 (5.64)	14.70 (2.98)	12.78 (4.82)	11.17 (6.10)	.06
Role-play+book^e^	12.19 (6.15)	13.78 (5.61)	13.91 (5.92)	14.60 (3.31)	13.00 (5.18)	.80
Best fine-tune+book^f^	11.70 (7.46)	13.33 (6.53)	15.15 (4.64)	13.64 (5.79)	12.17 (6.14)	.36

^a^Overall response score (the sum of 4 rating dimensions, with a maximum score of 20 representing the best performance).

^b^The fine-tuned model with 10,000 iterations.

^c^Role-play plus manual database retrieval.

^d^The best fine-tuned model plus manual database retrieval.

^e^Role-play plus book retrieval.

^f^The best fine-tuned model plus book retrieval.

### Performance Comparison: AI Model Versus Human Ophthalmologists

**Figure 4:**
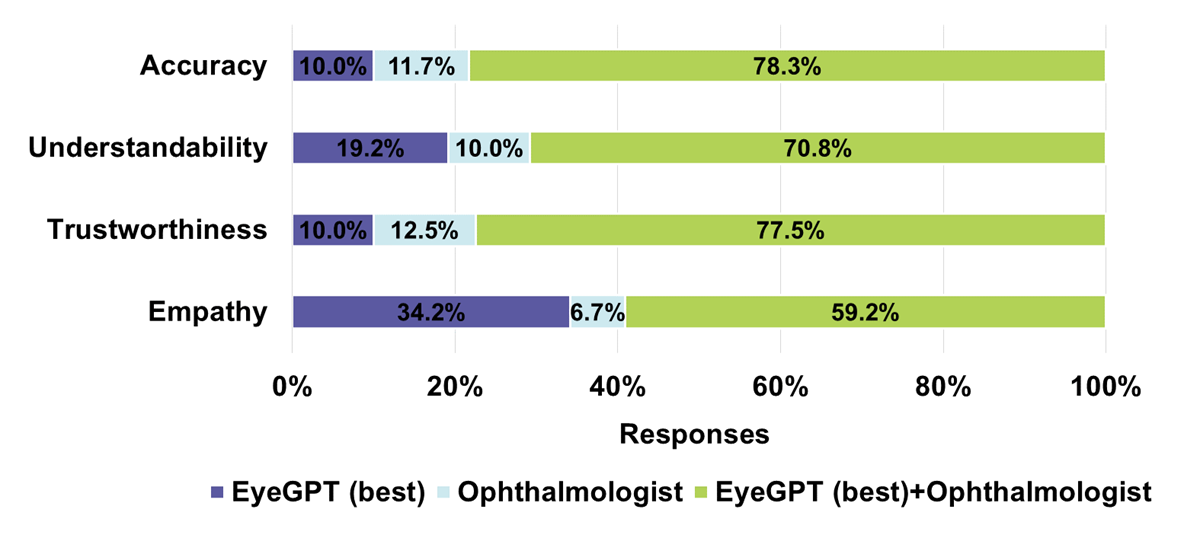
Percentage of answers ranked best by EyeGPT answers and ophthalmologists’ answers. EyeGPT(best): best fine-tune+book model.

In the human-machine best-ranked comparison, EyeGPT showed competitive capabilities, particularly in understandability and empathy. With the assistance of EyeGPT, human ophthalmologists’ performance was notably improved. Figure 4 summarizes the frequencies of answers generated by EyeGPT, unassisted ophthalmologists, or EyeGPT-assisted ophthalmologists, ranked as the best among the 3 candidate answers across 4 dimensions. Regarding understandability and empathy, the EyeGPT answers ranked best for 23 (19.2%) and 41 (34.2%) of the 120 questions, respectively, which were higher than those of ophthalmologists, which ranked best for 12 (10%) and 8 (6.7%) of the 120 questions. The answers provided by EyeGPT-assisted ophthalmologists were most frequently ranked as the best, at 85 (85/120, 70.8%) and 71 (71/120, 59.2%) for understandability and empathy, respectively; however, the accuracy and trustworthiness of EyeGPT answers were slightly lower than those by the ophthalmologists (accuracy: 12/120, 10% vs 14/120, 11.7%; trustworthiness: 12/120, 10% vs 15/120, 12.5%), highlighting areas for improvement. With the assistance of EyeGPT, the answers provided by the ophthalmologists excelled, ranking highest in accuracy and trustworthiness in 94 (78.3%) and 93 (77.5%) of the 120 questions, respectively. For illustrative examples of the best-ranked comparison, see Multimedia Appendix 11.

### Error Analysis: Areas for Improvement

The results of the error analysis are shown in Multimedia Appendix 12. Rater 1 identified 5 (5/120, 4.2%) QA pairs as containing unrelated information, 35 (35/120, 29.2%) as containing apparent factual errors, 23 (23/120, 19.2%) as having incomplete information, and 6 (6/120, 5%) exhibiting faulty logic. Rater 2 found 6 (6/120, 5%) QA pairs with unrelated information, 30 (30/120, 25%) with factual errors, 22 (22/120, 18.3%) with incomplete information, and 4 (4/120, 3.3%) demonstrating faulty logic. The inter-rater reliability, assessed using kappa values, was 0.905, 0.895, 0.699, and 0.792, respectively.

## Discussion

### Principal Findings

In this study, we integrated specialized ophthalmic knowledge into a general LLM using role-play, fine-tuning, and RAG methods, resulting in the development of EyeGPT for ophthalmology. In terms of accuracy, understandability, trustworthiness, and empathy, all fine-tuned models showcased remarkable improvements compared with the original model. Among them, the best fine-tune model exhibited the highest efficacy. Among the RAG strategies, the best fine-tune+book model emerged as the most capable. Subgroup analysis revealed that EyeGPT performed well in the category of common diseases and showed consistent performance across different users and domains. EyeGPT demonstrated competitive capabilities in understandability and empathy when compared with a human ophthalmologist. With the assistance of EyeGPT, the performance of the ophthalmologists was notably enhanced.

### Comparison With Prior Work

LLMs in health care raise concerns about inaccurate recommendations and fabricated information (hallucinations), which could lead to severe consequences. Previous studies have assessed the QA capabilities of existing LLMs in ophthalmology [11-13,15], highlighting the significance of augmenting LLMs with ophthalmic expertise. Our study achieved this by using 3 optimization methods: role-play, fine-tuning, and RAG. Role-playing helped position EyeGPT as an ophthalmologist, resulting in more professional responses, evidenced by the significantly increased accuracy, understandability, and trustworthiness. By setting the input role as the patient or student, the LLM’s response tended to be more compassionate and preaching, as reflected in the higher empathy scores than those with the original model. “To Cure Sometimes, To Relieve Often, To Comfort Always” is a well-known saying in medicine reminding us that providing care involves not only treating ailments but also offering relief and comfort to patients. Similarly, AI models should also embody empathy when assisting users, underscoring the importance of role-playing in developing medical AI assistants. Fine-tuning with publicly available real-world patient-doctor interactions further enhanced EyeGPT’s knowledge and performance. In addition, we observed that the reduction in evaluation loss with the validation set was consistent with the improvement in the model’s performance as evaluated by ophthalmologists. RAG is another way to make the LLM knowledgeable, particularly to reduce hallucinations. In previous studies, Zakka et al [10] developed Almanac, an LLM framework augmented with retrieval capabilities from curated medical resources for medical guidelines and treatment recommendations. In ophthalmology, Singer et al [41] used verified ophthalmology textbooks as source material, providing citations to address the trustworthiness and accuracy gaps in LLM responses to Ophthalmic Knowledge Assessment Program style queries. In this study, hallucination mitigation was also observed in the model enhanced by the manual database or books, reducing it by over 3.4% compared with the best fine-tune model. Among them, the best fine-tune+book model demonstrated the highest proportion of mitigating hallucinations and outperformed best fine-tune+database in all 4 aspects, which could potentially be attributed to the fact that the books surpassed the self-manual database regarding content richness and reference value.

Interestingly, we found no significant difference in performance between RAG and fine-tuned models. Fine-tuning is a popular approach but has limitations. One limitation is its dependence on specific formats of medical dialogue data, which are scarce and require validation and curation by medical professionals [2]. RAG overcomes these issues by directly leveraging authoritative external resources like textbooks, medical literature, or professional websites [42]. However, it is important to note that these optimization methods are not mutually exclusive. Our results demonstrated the combined effectiveness of fine-tuning and RAG, with the best-performing EyeGPT model obtained through integration. Furthermore, the data used for fine-tuning are publicly available and reliable, and the enhanced ophthalmic books are also openly accessible, rendering these strategies valuable references for future specific LLMs.

The health care environment is complex; therefore, it is essential to assess the performance of health care AI models in different scenarios [28]. Current research has primarily focused on evaluation for general questions [15], with limited studies on specific and rare diseases. Our study validated EyeGPT by analyzing its performance across various disease categories, demonstrating strong performance in common diseases but indicating room for improvement in special and rare diseases. Future improvements can be achieved by using high-quality data sets, specialized external knowledge resources, and exploring low-shot or few-shot learning. Additionally, we found that solely fine-tuned and RAG models were less informative for specialized medical student inquiries than simpler patient inquiries. The best-performing EyeGPT performed equally well for patient and student inquiries, suggesting that combining fine-tuning and RAG enhances LLM’s expertise in meeting the needs of both groups. Importantly, our evaluation set covers a wide range of question categories, from common diseases to rare diseases, and user roles encompassing patients and medical students, including disease descriptions, examinations, treatments, and more. By establishing multiple evaluation dimensions, including accuracy, understandability, trustworthiness, empathy, and hallucination, we aimed to provide a comprehensive reference framework for future ophthalmic specialized models.

Despite a growing global ophthalmologist workforce, limited-resource countries face a severe shortage of specialists [43]. EyeGPT has the potential to address this gap. Although its accuracy and trustworthiness are lower than those of human ophthalmologists, our findings show competitive capabilities in terms of understandability and empathy. This finding aligns with another study demonstrating the potential advantages of LLMs at enhancing efficiency and empathy in outpatient environments [44]. We attribute this to EyeGPT’s ability to patiently process large amounts of information and initiate and conclude conversations with consistent courtesy, unaffected by fatigue, emotions, or other factors. Although this may not be genuine in the human sense in high-demand scenarios, EyeGPT received higher empathy scores than human doctors who may, at times, provide brief responses or use complex medical jargon due to their level of medical knowledge, potentially leading to issues with poor understandability and empathy. However, the LLMs’ simplified expressions may overlook certain nuanced yet crucial medical information, leading to decreased accuracy and trustworthiness. The error analysis revealed that the main gap lies in factual inaccuracies and incomplete responses, highlighting the need to integrate more ophthalmic knowledge into the model and combine it with the professional expertise and experience of human doctors for comprehensive decision-making. Although LLMs cannot replace human professionals, they could serve as an auxiliary tool to enhance physicians' performance. In our ideal scenario, EyeGPT acts as a continuous, personalized assistant, providing guidance and clarification to patients throughout their care journey, without relying on physical queues or multiple face-to-face interactions with health care personnel. Additionally, EyeGPT can serve as an educational tool for medical students seeking immediate clarification on complex subjects. For example, EyeGPT may help primary doctors improve their decision-making ability and reduce diagnosis time.

### Limitations

Our study has several limitations. First, the current version of the model focuses on augmenting ophthalmic knowledge at the textual level. Future iterations should prioritize enhancing the model’s image interpretation capabilities [7,39,45], crucial for ophthalmology given its heavy reliance on multimodal imaging. Second, assessing the appropriateness of medical advice may be subjective and biased by grader opinion. More efforts could be achieved in the future, for example, by incorporating a broader spectrum of ophthalmic data and real-world feedback from users including medical students and patients. Last, a more secure application at this stage is using LLMs to assist physicians in their face-to-face consultations. This pilot study has initially validated its potential, and forthcoming research should aim to disseminate the findings more widely among the population.

### Conclusions

In conclusion, through role-playing, fine-tuning, and RAG, EyeGPT can potentially improve accuracy and efficiency in patient consultation and medical education. It may also be expected to increase access to high-quality medical consultations, especially for patients in underprivileged regions. We hope our study can make a good contribution to the current literature on ophthalmic AI assistants to provide an effective tool for enhancing health care.

## Appendix

Multimedia Appendix 1 Public datasets used in fine-tuning EyeGPT.

Multimedia Appendix 2 The specific list of keywords used in the public datasets filtering process.

Multimedia Appendix 3 The specific list of textbooks used in knowledge enhancement.

Multimedia Appendix 4 Sample of our manual database.

Multimedia Appendix 5 Specific diseases of question lists.

Multimedia Appendix 6

Multimedia Appendix 7 Tensorboard training logs of Finetune 3.

Multimedia Appendix 8 Statistical analysis of independent evaluations of 120 questions on the test set along four dimensions.

Multimedia Appendix 9 Inter-rater reliability analysis of 120 questions on the test set along four dimensions in independent evaluation.

Multimedia Appendix 10 Examples of generated answers with difference grating in independent evaluation.

Multimedia Appendix 11 Examples in best-ranked comparison.

Multimedia Appendix 12 Error analysis of the EyeGPT.

## Abbreviations

AI: artificial intelligence
FAISS: Facebook AI Similarity Search
LLM: large language model
LoRA: low-rank adaptation
QA: question-answering
RAG: retrieval-augmented generation
USMLE: United States Medical Licensing Examination

## Data Availability

The data sets generated during this study are available in the figshare repository at [46]. EyeGPT is available for use via [47].
