# Supplementary material for: EyeGPT for Patient Inquiries and Medical Education: Development and Validation of an Ophthalmology Large Language Model
Source: J Med Internet Res. 2024 Dec 11;26:e60063. doi: 10.2196/60063 (PMC11669878; doi:10.2196/60063)
Supplement: Multimedia Appendix 1 [file jmir_v26i1e60063_app1.pdf]

Multimedia Appendix 1. Public datasets used in fine-tuning EyeGPT.

Dataset	Original	Eye-specific
MedAlpaca	898199	56191
medical_meadow_cord19	821007	50214
medical_meadow_medical_flashcards	33955	1556
medical_meadow_medqa	10178	1737
medical_meadow_wikidoc	10000	945
medical_meadow_health_advice	8676	186
medical_meadow_wikidoc_patient_information	5942	576
medical_meadow_mmmlu	3787	118
medical_meadow_pubmed_causal	2446	39
medical_meadow_mediqa	2208	820
USMLE	10178	1705
MedMCQA	175890	19043
GenMedGPT-HealthCareMagic	117617	6980

USMLE=United States Medical Licensing Examination. MCQA=Multiple-Choice Question Answering. GPT=generative pre-trained transformer.
