# Supplementary material for: EyeGPT for Patient Inquiries and Medical Education: Development and Validation of an Ophthalmology Large Language Model
Source: J Med Internet Res. 2024 Dec 11;26:e60063. doi: 10.2196/60063 (PMC11669878; doi:10.2196/60063)
Supplement: Multimedia Appendix 2 [file jmir_v26i1e60063_app2.pdf]

Multimedia Appendix 2. The specific list of keywords used in the public datasets filtering process.

**Categories**	**Keywords**
Structure and Function	eye, sclera, retina, visual, vision, ophthal, cornea, optome, ocula, optic, conjunctiv, pupil, iris, macula, ocular lens, vitreous, choroid, fundus, fovea, tear film, aqueous humor, ciliary, oblique muscle, lacrimal, trabecular, meshwork.
Diseases and Symptom	myopia, strabis, amblyo, keratitis, uvei, hyperopia, cataract, pseudophakia, blindness, glaucoma, pterygium, keratoconus, retinopa, chorior, retinitis, iritis, amsler grid, chalazion, Stargardt, refractive, Lattice degeneration, episcle, scleritis, cycloplegic, trachoma, diplopia, aqueous flare, achromatopsia, dacryocystitis, nystagmus, ptosis.
Examination and Treatment	angiography, slit lamp, fluorescein, tonometry, electroretinogram, iridectomy, phacoemulsification, perimetry, schlemm, vitrectomy, pars plana, kerato, glasses, capsulotomy.
