# Supplementary material for: EyeGPT for Patient Inquiries and Medical Education: Development and Validation of an Ophthalmology Large Language Model
Source: J Med Internet Res. 2024 Dec 11;26:e60063. doi: 10.2196/60063 (PMC11669878; doi:10.2196/60063)
Supplement: Multimedia Appendix 3 [file jmir_v26i1e60063_app3.pdf]

Multimedia Appendix 3. The specific list of textbooks used in knowledge enhancement.

Book Title	Editors	Publication Date	Publisher	ISBN
Oxford Handbook of Ophthalmology	Alastair K. Denniston	2014	Oxford University Press	978-0199679980
Retina	Stephen J. Ryan	2013	Saunders	978-1455707379
Basic and Clinical Science Course, Section 02: Fundamentals and Principles of Ophthalmology	Lawrence M. Levine	2014-2015	American Academy of Ophthalmology	978-1615255566
Basic and Clinical Science Course, Section 03: Clinical Optics	Dimitri T. Azar	2014-2015	American Academy of Ophthalmology	978-1615255573
Basic and Clinical Science Course, Section 04: Ophthalmic Pathology and Intraocular Tumors	Robert H. Rosa Jr	2014-2015	American Academy of Ophthalmology	978-1615255580
Basic and Clinical Science Course, Section 05: Neuro-Ophthalmology	Rod Foroozan	2014-2015	American Academy of Ophthalmology	978-1615255597
Basic and Clinical Science Course, Section 06: Pediatric Ophthalmology and Strabismus	Gregg T. Lueder	2014-2015	American Academy of Ophthalmology	978-1615255603
Basic and Clinical Science Course, Section 07: Orbit Eyelids and Lacrimal System	John Bryan Holds	2014-2015	American Academy of Ophthalmology	978-1615255610
Basic and Clinical Science Course, Section 08: External Disease and Cornea	Robert W. Weisenthal	2014-2015	American Academy of Ophthalmology	978-1615255627
Basic and Clinical Science Course, Section 09: Intraocular Inflammation and Uvetis	Ramana S. Moorthy	2014-2015	American Academy of Ophthalmology	978-1615255634
Basic and Clinical Science Course, Section 10: Glaucoma	George A. Cioffi	2014-2015	American Academy of Ophthalmology	978-1615255641
Basic and Clinical Science Course, Section 11: Lens and Cataract	James C. Bobrow	2014-2015	American Academy of Ophthalmology	978-1615255658
Basic and Clinical Science Course, Section 12: Retina	Hermann D.	2014-2015	American Academy of	978-1615255665

and Vitreous	Schubert		Ophthalmology	
Basic and Clinical Science Course, Section 13: Refractive Surgery	M. Bowes Hamill	2014-2015	American Academy of Ophthalmology	978-1615255672
