# Supplementary material for: EyeGPT for Patient Inquiries and Medical Education: Development and Validation of an Ophthalmology Large Language Model
Source: J Med Internet Res. 2024 Dec 11;26:e60063. doi: 10.2196/60063 (PMC11669878; doi:10.2196/60063)
Supplement: Multimedia Appendix 4 [file jmir_v26i1e60063_app4.pdf]

Multimedia Appendix 4. Sample of our manual database.

Manual database

Disease: Myopia.

Epidemiology: The prevalence of myopia varies greatly between different populations and ethnic groups.

Risk factors: genetics, lifestyle factors (higher amounts of near-work activities, less outdoor activity).

Etiology: Myopia is a complex disease with a multi-factorial etiology. It is well documented that pathological non-syndromic high myopia and associated syndromic high myopia show evidence of familial inheritance; Although non-syndromic high myopia is most commonly inherited in an autosomal dominant pattern, multiple chromosomal loci have been identified which suggests genetic heterogeneity; High myopia is also a symptom of several multi-system complex diseases. The genetic mutations for these syndromes have been identified and the subsequent structural defects of the eye are most commonly related to connective tissue and retina. This type of myopia is only a small proportion of the overall myopic population and to date, there is no known isolated gene associated with physiologic myopia.

Classification|type: 1. Low myopia is -3.00 D or less; 2. Moderate myopia ranges between -3.25 to -6.00 D; 3. Severe myopia is anything above -6.00 D.

Characterized: The degree of non-pathologic myopia is usually minimal to moderate (< 6.00 diopters) and onset usually begins during childhood or adolescence; Pathologic myopia is usually defined as spherical equivalent > 6.00 diopters or axial length >26.5mm.

Pathology: 1. longer eyeball length than average ; 2. cornea, the clear covering in front of your eye, is too steep.

Disease complications: retinal detachment; choroid neovascularization; cataracts; glaucoma; retinal holes or tears; retinal detachments; chronic eyestrain and headaches; difficulty performing and interacting at school; impaired safety, especially while driving or operating machinery.

Diagnosis|Symptoms: Blurred vision when looking at faraway objects; Clear vision when looking at nearby objects; Eye strain; Squinting; Headaches.

Further examination: Refraction assessment; Eye health exam.

General treatment: wear glasses to see far away (low myopia); wear glasses full-time (moderate to severe myopia); bifocal or progressive eyeglasses (presbyopia (Nearsighted patients over 40)); Orthokeratology (Ortho-K); Multifocal Contact Lenses.

Treatment|Surgical intervention: LASIK: Laser-assisted in situ keratomileusis (LASIK) is the most common type of refractive surgery. During a LASIK procedure, your eye surgeon cuts a small flap in your cornea. They use a laser to reshape your corneal tissue, then replace the flap; LASEK: Laser-assisted subepithelial keratectomy (LASEK) is similar to LASIK. During LASEK, your surgeon creates a thin flap in the outermost layer of your cornea (epithelium). They reshape the outer layers of your cornea, then close the flap; PRK: Photorefractive keratectomy (PRK) involves completely removing the epithelium; Refractive Lens Exchange: This procedure removes the natural lens in your eye, which is very similar to cataract surgery; Phakic Intraocular Lens Implant: The main difference between this procedure and a refractive lens exchange is that your natural lens stays in place. Instead, the surgeon will place an artificial lens in front of your natural lens. Or, the surgeon may place the implant in front of the iris.
