# Supplementary material for: EyeGPT for Patient Inquiries and Medical Education: Development and Validation of an Ophthalmology Large Language Model
Source: J Med Internet Res. 2024 Dec 11;26:e60063. doi: 10.2196/60063 (PMC11669878; doi:10.2196/60063)
Supplement: Multimedia Appendix 5 [file jmir_v26i1e60063_app5.pdf]

Multimedia Appendix 5. Specific diseases of question lists.

**Disease Type**	**Disease**	**Question Count[a]**
Common	myopia	10
	glaucoma	10
	cataract	10
	diabetic retinopathy	10
Specialty	choroidal neovascularization	10
	central serous chorioretinopathy	10
	retinal detachment	10
	retinal vein occlusion	10
Rare	Best's disease	10
	morning glory syndrome	10
	Leber hereditary optic neuropathy	10
	Stickler syndrome	10

[a].Each character type includes one question from each of the five domains, leading to a total of 10 questions per disease. Questions for patients and medical students are based on different complexity and medical knowledge requirements.
