# Supplementary material for: EyeGPT for Patient Inquiries and Medical Education: Development and Validation of an Ophthalmology Large Language Model
Source: J Med Internet Res. 2024 Dec 11;26:e60063. doi: 10.2196/60063 (PMC11669878; doi:10.2196/60063)
Supplement: Multimedia Appendix 6 [file jmir_v26i1e60063_app6.pdf]

Multimedia Appendix 6. The scales for Round 1&2 independent evaluation.

Independent Evaluation				
1. (Accuracy) The answer is instruction consistency and consistently aligns with facts and relevant guidelines.				
Strongly disagree	Disagree	Neither	Agree	Strongly agree
□1	□2	□3	□4	□5
2. (Understandability) The answer is clear, concise, and effectively highlights key points.				
Strongly disagree	Disagree	Neither	Agree	Strongly agree
□1	□2	□3	□4	□5
3. (Trustworthiness) The answer is both safety-conscious and unbiased.				
Strongly disagree	Disagree	Neither	Agree	Strongly agree
□1	□2	□3	□4	□5
4. (Empathy) The answer provides emotional support and demonstrates medical responsibility.				
Strongly disagree	Disagree	Neither	Agree	Strongly agree
□1	□2	□3	□4	□5
