# Supplementary material for: EyeGPT for Patient Inquiries and Medical Education: Development and Validation of an Ophthalmology Large Language Model
Source: J Med Internet Res. 2024 Dec 11;26:e60063. doi: 10.2196/60063 (PMC11669878; doi:10.2196/60063)

Multimedia Appendix 7. Tensorboard training logs of Finetune 3. A. Training loss curve. B. Evaluation loss curve. C. Learning rate curve.

A.

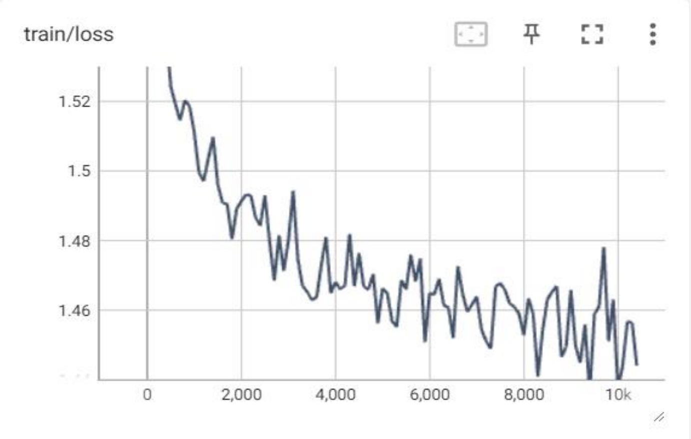

B.

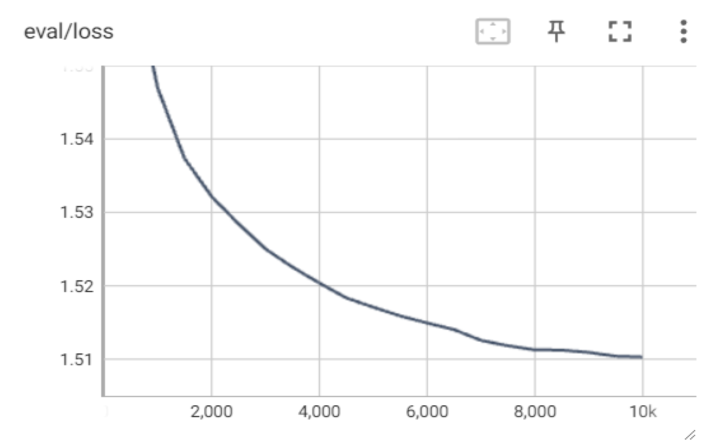

C.

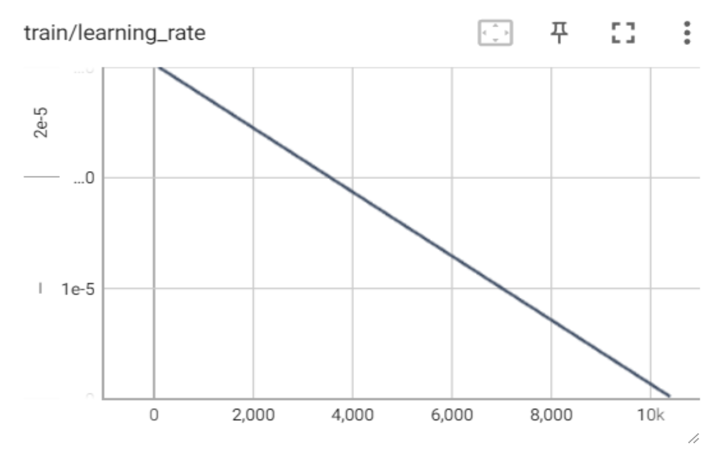

Supplement: Multimedia Appendix 7 [file jmir_v26i1e60063_app7.pdf]
