# Supplementary material for: EyeGPT for Patient Inquiries and Medical Education: Development and Validation of an Ophthalmology Large Language Model
Source: J Med Internet Res. 2024 Dec 11;26:e60063. doi: 10.2196/60063 (PMC11669878; doi:10.2196/60063)
Supplement: Multimedia Appendix 8 [file jmir_v26i1e60063_app8.pdf]

Multimedia Appendix 8. Statistical analysis of independent evaluations of 120 questions on the test set along four dimensions. A. Round 1. B. Round 2.

A

	**Accuracy**	**Understandability**	**Trustworthiness**	**Empathy**	**Total Score**	***P* value**[a]
**Original**	2.43 ± 1.17	2.25 ± 1.12	2.48 ± 1.38	2.15 ± 1.16	9.30 ± 4.42	**NA**
**Role-play**	3.17 ± 1.36	3.16 ± 1.45	3.34 ± 1.48	3.12 ± 1.46	12.79 ± 5.43	**<.001**
**Finetune 1**	3.10 ± 1.27	3.26 ± 1.44	3.34 ± 1.54	3.25 ± 1.46	12.95 ± 5.41	**<.001**
**Finetune 2**	3.09 ± 1.32	3.26 ± 1.43	3.33 ± 1.56	3.15 ± 1.48	12.83 ± 5.42	**<.001**
**Finetune 3**	3.38 ± 1.48	3.46 ± 1.48	3.50 ± 1.57	3.45 ± 1.47	13.79 ± 5.70	**<.001**

B

	**Accuracy**	**Understandability**	**Trustworthiness**	**Empathy**	**Total Score**	***P* value**[a]
**Best-finetune**	3.00 ± 1.33	3.30 ± 1.41	3.26 ± 1.49	3.52 ± 1.49	13.08 ± 5.43	NA
**Role-play+database**	3.18 ± 1.21	3.56 ± 1.43	3.44 ± 1.45	3.51 ± 1.35	13.69 ± 5.15	.35
**Best-finetune+database**	3.18 ± 1.14	3.56 ± 1.35	3.47 ± 1.36	3.51 ± 1.30	13.73 ± 4.83	.46
**Role-play+book**	3.32 ± 1.17	3.67 ± 1.29	3.63 ± 1.32	3.79 ± 1.22	14.41 ± 4.75	.08
**Best-finetune+book**	3.44 ± 1.19	3.88 ± 1.30	3.81 ± 1.30	4.01 ± 1.17	15.14 ± 4.64	**.001**

[a]Each round of each adjusted model and the base model (Llama2 or Best-finetune) was compared separately. P value is calculated with Mann-Whitney U test, with significant differences indicated in bold. The best score in each column is shown in red. Original (Llama2), Role-play (Original plus Role-play), Finetune 1-3 (Finetuned model versions 1-3 plus Role-play), Best-finetune (Finetune 3), Role-play+database (Role-play plus manual database retrieval), Best-finetune+database (the best finetuned model plus manual database retrieval), Role-play+book (Role-play plus book retrieval), Best-finetune+book (the best finetuned model plus book retrieval).
