# Supplementary material for: EyeGPT for Patient Inquiries and Medical Education: Development and Validation of an Ophthalmology Large Language Model
Source: J Med Internet Res. 2024 Dec 11;26:e60063. doi: 10.2196/60063 (PMC11669878; doi:10.2196/60063)
Supplement: Multimedia Appendix 9 [file jmir_v26i1e60063_app9.pdf]

Multimedia Appendix 9. Inter-rater reliability analysis of 120 questions on the test set along four dimensions in independent evaluation.

	**Round 1**			**Round 2**		
	Rater 1 Mean ± SD	Rater 2 Mean ± SD	Kappa	Rater 1 Mean ± SD	Rater 2 Mean ± SD	Kappa
**Accuracy**	3.09 ± 1.42	2.98 ± 1.37	0.711	3.03 ± 1.39	2.94 ± 1.33	0.861
**Understandability**	3.15 ± 1.52	3.00 ± 1.42	0.767	3.36 ± 1.56	3.26 ± 1.50	0.872
**Trustworthiness**	3.29 ± 1.62	3.10 ± 1.55	0.692	3.31 ± 1.59	3.19 ± 1.50	0.839
**Empathy**	3.16 ± 1.58	2.89 ± 1.45	0.726	3.60 ± 1.63	3.18 ± 1.46	0.611
