# Supplementary material for: EyeGPT for Patient Inquiries and Medical Education: Development and Validation of an Ophthalmology Large Language Model
Source: J Med Internet Res. 2024 Dec 11;26:e60063. doi: 10.2196/60063 (PMC11669878; doi:10.2196/60063)
Supplement: Multimedia Appendix 10 [file jmir_v26i1e60063_app10.pdf]

Multimedia Appendix 10. Examples of generated answers with difference grating in independent evaluation.

Question [Character]	Answer [Model]	Accuracy	Understandability	Trustworthiness	Empathy	Note
What are the differences between rhegmatogenous, tractional, and exudative retinal detachments? [Medical students]	Great question! Retinal detachment is a serious condition that can cause vision loss if not treated promptly. Here are the differences between rhegmatogenous, tractional, and exudative retinal detachments:<br>Rhegmatogenous retinal detachment occurs when there is a tear or hole in the retina, allowing fluid to pass through and separate the retina from the underlying tissue. This type of detachment is often caused by aging, injury, or diseases such as diabetes.<br>Tractional retinal detachment occurs when scar tissue forms on the surface of the retina, pulling it away from the underlying tissue. This can be caused by conditions such as uveitis, tumors, or inflammation.<br>Exudative retinal detachment occurs when fluid leaks out of the blood vessels in the retina, causing separation from the underlying tissue. This type of detachment is often caused by diseases such as age-related macular degeneration or diabetic retinopathy.<br>In all cases, prompt medical attention is necessary to prevent permanent vision loss. As an ophthalmologist, I	5	5	5	5	• The answer accurately explained the concepts of rhegmatogenous, tractional, and exudative retinal detachment, including their causes and mechanisms.<br>• The answer was concise and appropriately uses medical terminology, making it suitable for medical students studying the subject.<br>• The answer was reliable and does not contain any unsafe or biased information.<br>• The answer could address any further questions, demonstrating a caring attitude towards medical students seeking information.

	would perform a comprehensive eye exam to diagnose the type of retinal detachment and develop a treatment plan tailored to the individual patient's needs. Treatment may include surgery, laser therapy, or other interventions to repair the retina and prevent further vision loss.<br>I hope this information helps you understand the differences between these types of retinal detachments. Let me know if you have any other questions!<br>[Best fine-tune+book]					
My child has been diagnosed with morning glory syndrome. Is morning glory syndrome common?<br>[Patients]	Morning glory syndrome is a rare condition, affecting approximately 1 in 100,000 individuals worldwide. It is characterized by a congenital abnormality of the retina, where the blood vessels in the retina are abnormally dilated, giving the appearance of a morning glory flower. While it is uncommon, it is important to seek medical attention if you notice any changes in your child's vision or if they exhibit any signs of the condition, such as flashes of light or floaters. As an ophthalmologist, I understand the importance of early diagnosis and treatment to prevent long-term vision loss.<br>[Role-play+book]	4	4	4	4	● The answer mentioned the rarity of Morning Glory syndrome and the congenital abnormalities of the retina but incorrectly referred to it as vascular dilation instead of optic nerve dilation.<br>● The answer was clear, but it could be made more concise and easily understandable when explaining to the patient.<br>● The answer provided some safe and accurate medical information, but it omitted information about optic nerve damage and failed to provide warnings or

						preventive measures regarding patient safety and potential biases.<br>● The answer emphasized the importance of early diagnosis and treatment but lacked emotional resonance with parents and a deeper level of care and understanding.
Can hormones be used for CSC? [Medical students]	As an ophthalmologist, I understand that you are asking about the potential use of hormones in treating central serous chorioretinopathy (CSC). While there is some evidence to suggest that hormonal changes may play a role in the development of CSC, there is currently no established treatment that utilizes hormones to manage the condition.<br>[Best fine-tune+database]	3	3	3	3	● The answer addressed the uncertainty of using hormone therapy for CSC, but it did not specifically explain the necessity of discontinuing hormone use.<br>● The answer was concise and clear, but there is room for improvement in emphasizing key points.<br>● The answer failed to mention the safety risks associated with the use of hormones, which is an important consideration. Mishandling can potentially pose safety risks to clinical treatment.<br>● Additional background information or recommendations about CSC could be

						provided to meet the knowledge needs of medical students.
Is Leber hereditary optic neuropathy inherited? [Patients]	Leber hereditary optic neuropathy (LHON) is a rare genetic disorder that affects the optic nerve and can cause vision loss. It is caused by mutations in the genes that code for the proteins involved in the metabolism of the amino acid histidine. These mutations are inherited in an autosomal dominant pattern, which means that a single copy of the mutated gene is enough to cause the condition. This means that if one of your parents carries the mutated gene, you have a 50% chance of inheriting the mutated gene and developing LHON. However, it's important to note that not everyone who inherits the mutated gene will develop LHON, and some people may not develop any symptoms until later in life. Additionally, LHON can also be caused by acquired mutations, which can occur due to various factors such as viral infections or exposure to certain toxins. [Finetune 1]	2	2	2	2	● The answer only indicated the hereditary nature of LHON but did not accurately describe the genetic pattern of LHON.<br>● The answer's structure was relatively clear, but it was not concise. Complex terminology may make it difficult for ordinary readers to understand.<br>● The answer included inaccurate key medical information, which may mislead users seeking accurate information.<br>● The answer did not emphasize the importance of timely medical and genetic counseling, lacking care and understanding for the patients.
What characterizes PACG? [Medical students]	Here are some key characteristics of PACG:<br>1. **Deck-building: ** Players start with a small deck of cards and must strategically add new cards to their deck throughout the game to improve their chances of winning.	1	1	1	1	This answer did not correctly recognize the true meaning of PACG, resulting in the entire answer being completely unrelated to medicine.

2. **Hand management: ** Each player has a limited number of hands they can play each turn, and they must carefully choose which cards to play and when.
3. **Card combinations: ** Cards can be combined in various ways to create powerful combinations and strategies.
4. **Interaction: ** Players can interact with each other in various ways, such as by playing cards on each other's turns or by using special abilities to disrupt their opponents' plans.
5. **Luck vs. strategy: ** While luck can play a role in the game; it is ultimately a game of strategy and skill. Players must carefully plan their deck-building and card-playing strategies to come out on top.
6. **Replay ability:** With a large deck of cards and a variety of playstyles, each game of PACG can be different and offer new challenges and opportunities for strategic play.
7. **Scalability: ** PACG can be played with 2-4 players, making it a great option for groups of any size.
8. **Easy to learn: ** While the game has a lot of depth and complexity, it is relatively easy to learn and can be taught to new players in a short amount of time.
9. **Accessible: ** PACG is a game that can be enjoyed by players of all skill levels and ages, making it a great option for families and casual gamers.

	10. **Expansions: ** There are many expansions available for PACG, which offer new cards, mechanics, and gameplay elements to keep the game fresh and exciting.<br>By understanding these characteristics, you should have a better idea of what to expect when playing PACG and how to approach the game."<br>[Original]	
