# Supplementary material for: EyeGPT for Patient Inquiries and Medical Education: Development and Validation of an Ophthalmology Large Language Model
Source: J Med Internet Res. 2024 Dec 11;26:e60063. doi: 10.2196/60063 (PMC11669878; doi:10.2196/60063)
Supplement: Multimedia Appendix 12 [file jmir_v26i1e60063_app12.pdf]

Multimedia Appendix 12. Error analysis of the EyeGPT.

	Rater 1 N (%)	Rater 2 N (%)	Kappa
**Unrelated information**	5 (4.2%)	6 (5.0%)	0.905
**Factual error**	35 (29.2%)	30 (25.0%)	0.895
**Incomplete information**	23 (19.2%)	22 (18.3%)	0.699
**Faulty logic**	6 (5.0%)	4 (3.3%)	0.792
